# A heat and moisture-exchanging mask impairs self-paced maximal running performance in a sub-zero environment

**DOI:** 10.1007/s00421-021-04666-9

**Published:** 2021-03-29

**Authors:** Alasdair S. Tutt, Hampus Persson, Erik P. Andersson, Mats Ainegren, Nikolai Stenfors, Helen G. Hanstock

**Affiliations:** 1 Department of Health Sciences, Swedish Winter Sports Research Centre, Mid Sweden University, Studentplan 4, 831 40 Östersund, Sweden; 2 Unit of Medicine, Department of Public Health and Clinical Medicine, Umeå University, Umeå, Sweden; 3 Faculty of Health Sciences, School of Sport Sciences, UiT The Arctic University of Norway, Tromsø, Norway; 4 Department of Quality Management and Mechanical Engineering, Sports Tech Research Centre, Mid Sweden University, Östersund, Sweden

**Keywords:** Cold environment, Cross-country skiing, Exercise-induced bronchoconstriction, NIRS, Winter sports

## Abstract

**Purpose:**

Heat-and-moisture-exchanging devices (HME) are commonly used by endurance athletes during training in sub-zero environments, but their effects on performance are unknown. We investigated the influence of HME usage on running performance at − 15 °C.

**Methods:**

Twenty-three healthy adults (15 male, 8 female; age 18–53 years; $\dot{V}{\text O}_{2peak}$ men 56 ± 7, women 50 ± 4 mL·kg^−1^·min^−1^) performed two treadmill exercise tests with and without a mask-style HME in a randomised, crossover design. Participants performed a 30-min submaximal warm-up (SUB), followed by a 4-min maximal, self-paced running time-trial (TT). Heart rate (HR), respiratory frequency (*f*_R_), and thoracic area skin temperature (*T*_sk_) were monitored using a chest-strap device; muscle oxygenation (SmO_2_) and deoxyhaemoglobin concentration ([HHb]) were derived from near-infra-red-spectroscopy sensors on *m. vastus lateralis*; blood lactate was measured 2 min before and after the TT.

**Results:**

HME usage reduced distance covered in the TT by 1.4%, despite similar perceived exertion, HR, *f*_R_, and lactate accumulation. The magnitude of the negative effect of the HME on performance was positively associated with body mass (*r*^2^ = 0.22). SmO_2_ and [HHb] were 3.1% lower and 0.35 arb. unit higher, respectively, during the TT with HME, and *T*_sk_ was 0.66 °C higher during the HME TT in men. HR (+ 2.7 beats·min^−1^) and *T*_sk_ (+ 0.34 °C) were higher during SUB with HME. In the male participants, SmO_2_ was 3.8% lower and [HHb] 0.42 arb. unit higher during SUB with HME.

**Conclusion:**

Our findings suggest that HME usage impairs maximal running performance and increases the physiological demands of submaximal exercise.

## Introduction

Athletes undertaking training and competition in winter endurance sports, such as cross-country skiing and biathlon, are frequently exposed to extended durations of moderate-to-maximal-intensity exercise in sub-zero temperatures. Although sub-zero temperatures are associated with decreased performance in cross-country skiing (Lindberg et al. 2012; Wiggen et al. 2016), the minimum temperature allowed by the International Ski Federation in cross-country skiing competitions is − 20 °C, and so, athletes in these sports are prepared to train and compete in ambient temperatures that may not be favorable for optimal performance. High-volume training in sub-zero conditions may increase the likelihood of developing exercise-induced asthma (EIA), which may have long-term effects on health and performance (Sue-Chu 2012). Repeated provocation of the airways in cold climates may cause inflammation, injury, and edema, and underpin the development of EIA (Sue-Chu 2012). Strategies to decrease the incidence of cold-induced airway obstructions could play a key role in improving the lung function of athletes, but it is important that any preventive strategy does not interfere with the potential to achieve optimal performance.

The prevalence of asthma in cross-country skiers has been estimated at 23%, but may vary depending on the diagnostic criteria used (Mäki-Heikkilä et al. 2020). In Sweden, the prevalence of self-reported, physician-diagnosed asthma has been recently reported at 9% (Borna et al. 2019). Using similar criteria, the prevalence of asthma in Swedish adolescent cross-country skiers has been reported at up to 35% in Sweden (Norqvist et al. 2015; Eriksson et al. 2018), with circa 20–25% of athletes using asthma medications (Eriksson et al. 2018), and onset frequently occurring during adolescence (Eriksson et al. 2018). Furthermore, the incidence rate of self-reported, physician-diagnosed asthma among elite cross-country skiers has been estimated at 61/1000 person-years (Irewall et al. 2020) compared to around 4/1000 person-years in the Swedish general population (Ekerljung et al. 2008). Prevalence is also higher in women (Eriksson et al. 2018) which could be related to their smaller stature, whereby narrower airways may exacerbate shear stress on the airway lining at high rates of ventilation (Kennedy et al. 2019).

Cold environments exacerbate exercise-induced bronchoconstriction (EIB, defined as a > 10% decrease in FEV_1_) in asthmatic participants (Sandsund et al. 1997; Stensrud et al. 2007). However, non-asthmatic individuals can also experience subclinical reductions in FEV_1_ during exercise in cold climates (Kennedy and Faulhaber 2018). In particular, short-duration, near-maximal exercise in cold environments and competition-like settings has been linked to a high incidence of EIB during screening of winter athletes (Heir and Larsen 1995; Wilber et al. 2000; Kennedy et al. 2019). Seasonal increases in airway inflammatory biomarkers have recently been highlighted in winter endurance athletes without asthma (Kennedy et al. 2016; Kurowski et al. 2018), while respiratory symptoms, including post-exercise cough following training and competition in cold climates are also commonly reported (Kennedy et al. 2016; Kennedy and Faulhaber 2018; Sjöström et al. 2019). Thus, asthmatic and non-asthmatic athletes alike could potentially benefit from preventive strategies to maintain normal respiratory function during exercise in sub-zero temperatures. Prescription of asthma medication is not appropriate for non-asthmatic athletes and neither does it address the underlying causes of bronchoconstriction, that is, cooling and drying of the airway mucosa and damage to the airway epithelium.

Non-pharmaceutical preventive measures such as heat-and-moisture exchanger devices have been in use for at least three decades, with large variations in fit, materials and design (Hanstock et al. 2020). A heat-and-moisture exchanger device (HME) is effective in negating EIB during exercise in the cold (Millqvist et al. 1995, 2000; Beuther and Martin 2006; Frischhut et al. 2020). However, athletes in cross-country skiing and biathlon competitions seem to prefer facial coverings when competing in sub-zero conditions such as taping of the forehead, nose and cheeks, and thin neck warmers that can easily be moved to adjust coverage during competition. These coverings negate some of the facial cooling effect which may exacerbate bronchoconstriction (Koskela and Tukiainen 1995) but are not optimal solutions for warming and humidifying inspired air. The reasons for the lack of HME use by athletes in competitions are unclear but could reflect a perception of, or actual impairments to performance.

From the available data, it is reasonable to hypothesise that among healthy individuals, HME usage would facilitate optimal performance during short, high-intensity exercise in a sub-zero environment. However, it is equally reasonable to expect that a combination of discomfort, increased dead space and accumulation of ice in the filter could increase resistance to breathing, physiological strain or lead to athletes adopting a more conservative pacing strategy, each of which could impair maximal performance. Awareness of any effects of an HME on exercise performance in a sub-zero climate is, therefore, an important prerequisite prior to recommending HME usage in competition. To our knowledge, only one study has investigated the effects of an HME on physiological variables during high-intensity exercise in healthy individuals. Frischhut et al. (2020) reported improvements in lung function and lower perceived exertion in healthy athletes during intense exercise using an HME at a temperature of − 20 °C. HMEs may also have a positive effect on sprint performance in healthy subjects when worn between work periods in cold conditions (Seifert et al. 2017). However, no previous studies have assessed the effects of an HME on self-paced maximal endurance performance in a cold climate.

The aim of the present study was therefore to investigate the effect of a heat-and-moisture-exchanging mask on distance covered during a self-paced 4-min running time-trial (TT), performed by healthy individuals in a sub-zero environment. Secondary aims were to investigate the effects of an HME on physiological responses to submaximal and maximal TT exercise in a sub-zero environment, and to investigate potential sex differences in the effect of the HME on physiological responses and TT performance.

## Methods

### Participants

Twenty-three healthy, trained adult participants, aged 18–53 years, gave written, informed consent to participate in the study (15 male and 8 female; characteristics in Table 1). Participants’ health status was screened by a medical doctor prior to inclusion. Exclusion criteria included diagnosed asthma or airborne allergies, recent respiratory illness, and having ever been a regular smoker. All participants were accustomed to performing endurance training in sub-zero temperatures; six participants were currently competing at national or international level in winter sports (e.g., cross-country skiing, skijoring), while 17 participated at a recreational level. The study was performed in May and June, and thus, participants had no recent exposure to sub-zero climates.

**Table 1:** Participant characteristics as obtained during the familiarisation trial

	Men	Women	All
Age (years)	33 ± 9	28 ± 6	31 ± 8
Height (cm)	180 ± 7	168 ± 6	176 ± 9
Weight (kg)	77 ± 10	62 ± 5	72 ± 11
Maximum HR (beats·min^−1^)	190 ± 6	193 ± 4	191 ± 5
$\dot{V}{\text O}_{2peak}$ (L·min^−1^)	4.3 ± 0.6	3.1 ± 0.2	3.9 ± 0.8
$\dot{V}{\text O}_{2peak}$ (mL·kg^−1^·min^−1^)	56 ± 7	50 ± 4	54 ± 7
*V*_Epeak_ (L·min^−1^)	165 ± 22	111 ± 8	146 ± 32
RER_peak_	1.10 ± 0.08	1.17 ± 0.09	1.12 ± 0.08
RPE	18.7 ± 0.7	19.0 ± 0.6	18.8 ± 0.7
La^-^^a^ (mmol·L^−1^)	11.5 ± 2.9	10.4 ± 2.2	11.1 ± 2.7
*N* with ≥ 3 criteria for attainment of *V*O_2peak_	11/15	7/8	18/23
Distance covered in TT^b^ (m)	971 ± 97	857 ± 71	931 ± 104
Speed^b^ at 65% $\dot{V}{\text O}_{2peak}$ (km·h^−1^)	8.4 ± 1.1	7.4 ± 1.4	8.0 ± 1.3
Speed^b^ at 70% $\dot{V}{\text O}_{2peak}$ (km·h^−1^)	9.0 ± 1.2	8.0 ± 1.3	8.7 ± 1.3
Speed^b^ at 75% $\dot{V}{\text O}_{2peak}$ (km·h^−1^)	9.7 ± 1.3	8.6 ± 1.2	9.3 ± 1.4
Speed^b^ at 90% $\dot{V}{\text O}_{2peak}$ (km·h^−1^)	11.7 ± 1.6	10.5 ± 1.1	11.3 ± 1.6

Data are mean ± SD

RER_peak_: peak respiratory exchange ratio, RPE: Borg’s 6–20 rating of perceived exertion, TT: 4-min treadmill running time-trial (self-paced), $\dot{V}{\text O}_{2peak}$: peak oxygen uptake, *V*_Epeak_: peak minute ventilation

^a^2 min post-TT

^b^Performed at 4% treadmill gradient

### Study design and experimental set-up

Participants performed a familiarisation test followed by two experimental trials, with and without HME, in a randomised, crossover design. On each visit, participants completed a graded submaximal treadmill running protocol followed by a 4-min, self-paced, treadmill running TT. The TT was chosen for its high test–retest reliability and ecological validity as a measure of running performance (McGawley 2017), and is similar in duration to sprint races within cross-country skiing (Sandbakk et al. 2011). Performance was defined as distance covered during the TT.

All trials were carried out in sub-zero conditions in an environmental chamber, on a motorised treadmill with the capability for self-steered speed control (Rodby 2700E, Rodby Innovation AB, Vänge, Sweden). The self-steering system (Rodby Control System 2.0) utilised two independent infra-red sensors mounted to the front of the treadmill, that detected participants’ distance from the sensor. Speed remained stable in a fixed central 70 cm zone on the treadmill. By moving closer to the sensor into the 50 cm acceleration zone, the participant could accelerate at a graded rate of up to 1 km·h^−1^·s^−1^, and by moving backwards out of the no-change zone, the speed would decrease at the same rate. The system was used to deliver the TT during each trial. To control for differences in the initial acceleration, participants were instructed to start the test by standing at the front of the acceleration zone and to remain there for the first 10 s of the test.

Trials were conducted at least 72 h apart to avoid crossover effects, and at the same time of day to negate the influence of circadian rhythms. Participants were requested to not perform any strenuous training for 48 h before each trial, and to repeat a similar routine with regard to meal timing and composition before each visit. Participants were also instructed to avoid caffeine on the day of each trial, and alcohol for 24 h beforehand.

Clothing was recommended as a lightweight hat/headband, a fabric neck warmer, gloves, light windproof jacket over a technical base layer, and tights or pants. Additional base layering was optional. Layering changes were allowed during rest periods, but only one participant did so. The neck warmer was not allowed to cover the face, so as not to interfere with facial cooling upon cold exposure, that may influence lung function (Koskela and Tukiainen 1995).

### Familiarisation protocol

**Fig. 1:**
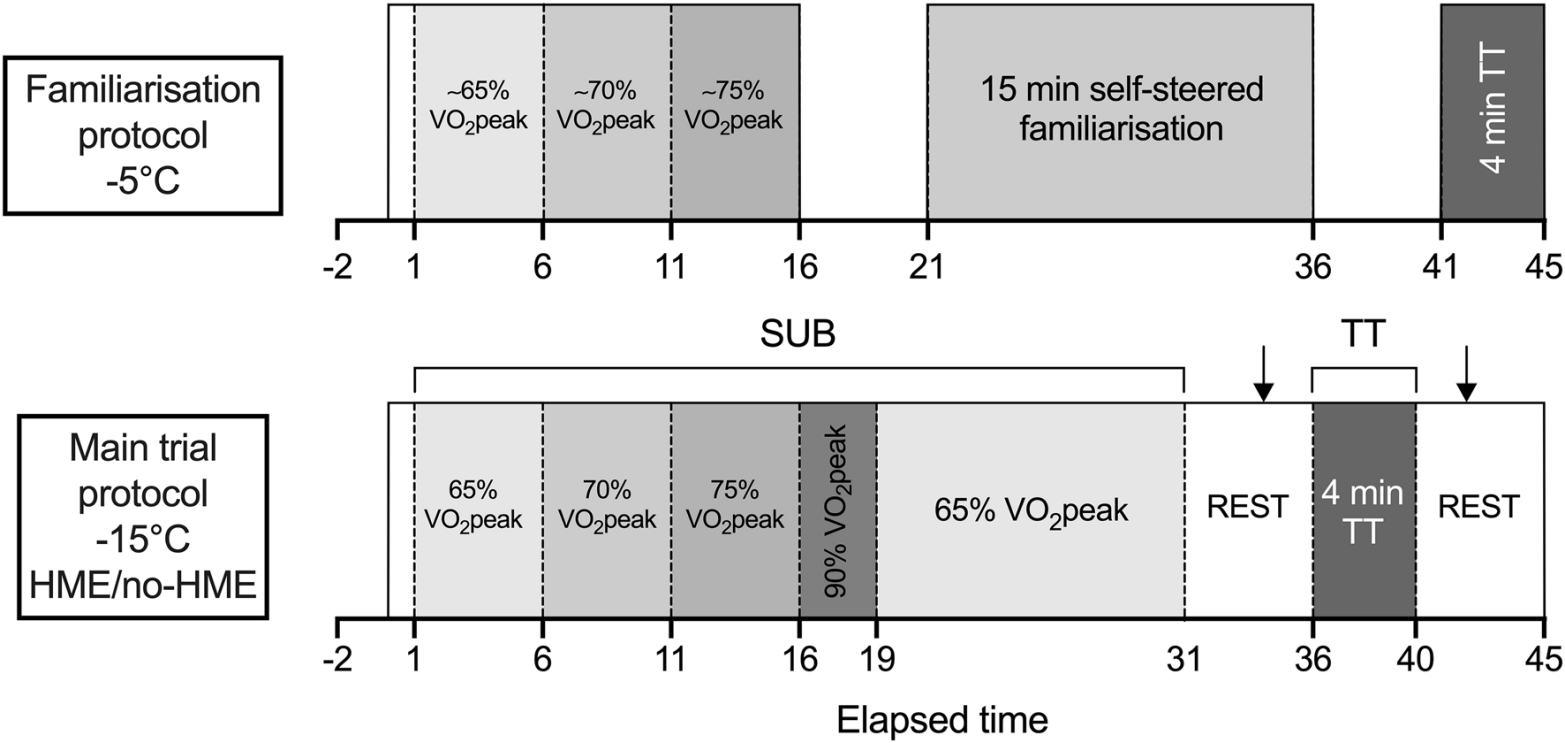
Protocol schematic diagram for familiarisation and main experimental trials. Solid boxes indicate time spent inside the environmental chamber. Arrows indicate blood lactate sampling. SUB: 30 min submaximal exercise bout; TT: 4-min self-paced running time-trial. The HME trial was performed in − 15 °C while wearing the HME continuously, whereas the no-HME trial was performed in − 15 °C with no facial covering

Participants performed a familiarisation running protocol (Fig. 1) at − 5 °C in the environmental chamber, at a gradient of 4%, while wearing a portable breath-by-breath metabolic cart system (Metamax 3B, Cortex Biophysik, Leipzig, Germany). Pilot work indicated that a temperature of − 5 °C was more appropriate for the familiarisation trial than − 15 °C to avoid freezing of moisture in the sampling tubes. The familiarisation protocol consisted of three 5-min submaximal stages to estimate target intensities for the SUB stages of the experimental protocol using the linear regression equation from the speed–oxygen uptake (*V*O_2_) relationship. *V*O_2peak_ was estimated based on participants’ physical activity level, previous race results, and/or previous laboratory test results. The speeds corresponding to oxygen uptake demands of 65, 70, and 75% of participants’ *V*O_2peak_ were estimated based on an assumed fixed gross oxygen cost of 0.249 mL·kg^−1^·m^−1^ for uphill running at a 4% gradient as based on the average value from previous (unpublished) laboratory data. The speeds (m·min^−1^) at the corresponding exercise intensities could then be calculated by dividing the intensity specific *V*O_2_ (mL·kg^−1^·min^−1^) with the gross oxygen cost (mL·kg^−1^·m^−1^) and converted to a speed in km·h^−1^. After a 5-min rest outside the chamber (to prevent freezing of moisture in the sampling line), participants were instructed to both familiarise themselves with the system and warm-up for the TT during a 15-min self-paced exercise bout. After a further 5-min rest, participants performed the 4-min TT as a maximal effort. *V*O_2peak_ was defined as the highest 20-s moving average during the TT. Criteria for attainment of peak oxygen uptake were respiratory exchange ratio ≥ 1.15, lactate concentration (La^-^) ≥ 8.0 mmol·L^−1^, Borg RPE score of 19 or 20, peak HR within 10 beats·min^−1^ of age-predicted maximum HR (211 − 0.64 × age; Nes et al. 2013), and attainment of a *V*O_2_ plateau (McGawley 2017). All participants obtained at least one of these criteria, and 18 of 23 attained ≥ 3 criteria, which is a comparable proportion to a previous study using a treadmill TT protocol (McGawley 2017). The equation for the linear regression between speed and submaximal *V*O_2_, based on the final-minute average from each stage, together with measured *V*O_2peak_, was used to revise submaximal speeds ahead of the main experimental trials.

### Experimental protocol

Participants performed the two experimental trials in the environmental chamber set to − 15 °C, with and without HME, in a randomised order. The HME device was selected based on a survey of students at Swedish high schools with regional cross-country ski programs where over 80% of HME users used products from the Airtrim brand (Stenfors et al. 2020). Thus, participants used an Airtrim mask (Vapro AB, Västerås, Sweden) with a Sport filter during the HME trial, with an inspiratory and expiratory resistance of 2.3 Pa·L^−1^·s^−1^ (Ainegren et al. 2020).

Temperature and relative humidity in the chamber were − 14.9 ± 0.2 °C and 69.5 ± 5.1% during the HME trial, and − 15.0 ± 0.1 °C and 70.2 ± 4.1% during the no-HME trial. Absolute humidity was 1.29 ± 0.09 and 1.30 ± 0.07 g·m^−3^, respectively (Sjöström et al. 2019). Dehumified air (20.9% O_2_) was delivered to the chamber at a rate of 1500 L·min^-1^ (K2-1500, Hypoxico Inc, Gardiner, NY, USA).

The protocol began with a 30-min, graded submaximal warm-up, performed at speeds between 65 and 90% *V*O_2peak_ (Fig. 1). The warm-up was designed to replicate a traditional long warm-up for a sprint cross-country skiing (Solli et al. 2020), with fixed workloads to allow for replication between trials. After a 5-min standing rest inside the chamber, participants performed the TT. Participants were instructed to perform a maximal effort, but no feedback or encouragement was provided during the TT besides remaining time at 3, 2, and 1 min, 30 and 15 s. Borg 6–20 rating of perceived exertion (RPE) was recorded 1 min prior to the end of each SUB stage from 65 to 75% and at the end of the 90% stage and TT. Participants recovered in the chamber for 5 min after conclusion of the TT.

Upon completion of the trial, participants completed a brief questionnaire to evaluate perceptions of how their performance and comfort were affected by the cold environment and use of the HME. The questionnaire contained three scale questions and a space for qualitative comments. The first question was “How would you rate your performance in the time-trial today?” (Q1). The second question was phrased differently for each trial, where in the no-HME trial, participants were asked: “To what extent do you think the cold affected your performance today, compared to if you were wearing a mask in the same temperature?” (Q2_C_), whereas in HME, the question was: “To what extent do you think wearing a mask affected your performance today, compared to if you had not worn the mask?” (Q2_H_). Each question was scored on a 7-point Likert scale where items ≤ 3 reflected negative perceptions, 4 = neutral, and ≥ 5 were positive perceptions. Scores for Q2_C_ were reversed before analysis. The third question asked the participants “How often do you use a breathing mask for your own training in winter when the temperature is below (a) 0 °C, (b) − 10 °C, and (c) − 20 °C?” (Q3) with answers 0 = “never”, 1 = “occasionally”, 2 = “sometimes”, 3 = “most of the time”, and 4 = “all the time”.

### Physiological measurements

Heart rate (HR), respiratory frequency (*f*_R_), and skin temperature (*T*_sk_) were measured using a chest harness device (LifeMonitor, Equivital, Cambridge, UK). HR was derived from a 2-lead electrocardiogram, *f*_R_ from an expansion sensor in the chest harness, and *T*_sk_ was measured at the left lateral thoracic region from an infra-red sensor embedded in the sensor module attached to the chest strap. Muscle oxygen saturation (SmO_2_, also known as the tissue saturation index or TSI) and total haemoglobin concentration ([THb]) were derived from two near-infra-red spectroscopic (NIRS) sensors placed on the belly of the left and right *m. vastus lateralis* (MOXY monitor, Fortiori Design, Hutchinson MN, USA). SmO_2_ represents the balance between oxygen delivery and extraction in the targeted muscle, whilst THb is indicative of local blood flow and can be used in combination with SmO_2_ to calculate local oxy- and deoxyhaemoglobin/myoglobin concentrations (Ferrari et al. 2011). Sensor placement was noted as the distance from the femoral lateral epicondyle (13–22 cm) and participants maintained a mark of the placement between experimental trials.

Fingertip capillary blood samples were obtained 2-min pre- and post-TT using a 1.6 mm safety lancet into 20 µL capillary tubes, that were haemolysed immediately and analysed for La^–^ the same day (Biosen 5140, EKF diagnostic GmbH, Magdeburg, Germany).

### Data preprocessing

Preprocessing of trace data (HR, *f*_R,_ *T*_sk_, and NIRS data) was performed using the Python 3 programming language. First, data were filtered for extreme and null values by replacing values deviating > 2 SD from the mean of the surrounding 6 data points with the local mean (Van Der Zwaard et al. 2016) and visually inspected for erroneous regions. Then, data were averaged to 30- and 10-s epochs for the submaximal exercise bout and 4-min TT, respectively.

Data loss sometimes occurred due to technical issues such as signal dropout, overwrite, or movement artefacts. Where a complete trial’s worth of data was lost for a given variable, data from the participants’ other trial were also excluded from further analyses of that variable.

There was a complete bilateral loss of NIRS data in three trials (*n* = 3 participants excluded) and partial unilateral loss/erroneous readings (identified through visual inspection) in a further seven SUB bouts and five TTs, leading to one more participant being excluded from the analysis of SUB. Where an NIRS trace was lost or erroneous for only one sensor, then only the data from the other device on the contralateral leg were included for analysis in both trials; otherwise, the mean value from the two sensors was used. Relative deoxygenated haemoglobin and myoglobin concentration ([HHb]) was then derived from [THb] and SmO_2_: [THb] − ([THb] × (SmO_2_/100)) (Ferrari et al. 2011). Peak deoxygenation was defined as the difference between maximum and minimum SmO_2_ values during the 4-min TT.

There was a complete loss of HR data in three trials from four participants and partial loss of HR data during SUB in one further trial, leading to *n* = 19 (SUB) and *n* = 20 (TT) participants’ data being analysed. *f*_R_ and *T*_sk_ data were lost from one full trial. Three participants across four trials reached the maximal *f*_R_ detectable by the device of 70 breaths·min^−1^; these data were retained for analysis.

### Statistical analysis

A statistical power calculation was performed a priori using data from McGawley (2017). To achieve statistical significance for a mean difference in performance of 1.8%, equivalent to one coefficient of variation (CV), and thus, outside the normal range of performance for a 4-min treadmill running TT, the estimated Cohen’s *d* effect size was 0.67 (Between-subject CV = 8.4%, ICC = 0.95). With alpha = 0.05 and power = 0.8, a sample size of 20 participants was estimated to be required.

Statistical analyses were performed using the jamovi R interface (jamovi version 1.2, the jamovi project) and GraphPad Prism (v8, GraphPad Software, San Diego, CA, USA). Performance and physiological variables were visually inspected for normality and are expressed as mean ± SD. Significance was set at an alpha level of 0.05; *p* = 0.05–0.1 was considered a trend. Two-way mixed ANOVA was used to test for effects of HME and sex on distance covered, peak deoxygenation, maximal RPE, and lactate accumulation, and partial eta squared effect sizes calculated for these analyses )$\eta_{{\text{p}}}^{2}$). Linear regressions were performed to explore relationships between anthropometric and physiological data and the effect of the HME on distance covered. Linear mixed models were used to investigate trace variable responses to exercise; for the TT, all the data were used, whereas for SUB, 60-s averages taken 30 s before the end of each stage were included in the analysis. Fixed effects were elapsed time, trial, sex, and two- and three-way interactions between them, and each model included a random effect for participant. Models were further refined by removing non-significant interactions. 95% confidence intervals of the difference (95% CI) between trials are presented for pairwise comparisons and fixed-effects parameter estimates in the linear mixed models. Bonferroni post hoc tests were used to explore significant interactions. For clarity, main effects of time are not reported, and where there were no significant sex × trial interactions, participants’ data are reported for the whole cohort. Questionnaire scale items were analysed using the Wilcoxon test and thematic analysis was performed on the comments.

## Results

### Effect of the HME on performance

**Fig. 2:**
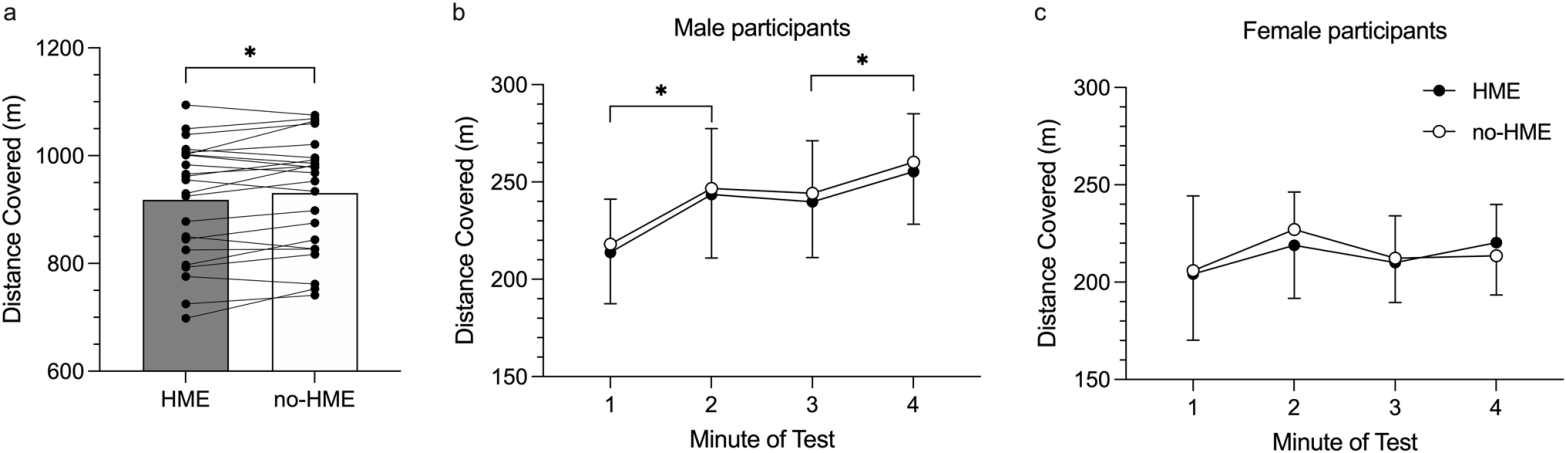
**a** Effect of HME usage on 4-min self-paced running time-trial performance in − 15 °C (*n* = 23). Bars show mean distance for each trial, with individual responses overlaid. *Difference in distance covered between trials, *p* < 0.05. **b**, **c** Effect of HME usage during exercise in − 15 °C on pacing strategy in male (**b**, *n* = 15) and female participants (**c**, *n* = 8). Data are mean ± SD. *Difference between distance covered in adjacent minutes, *p* < 0.05

Use of an HME during the TT resulted in a 13 m (− 1.4%) reduction in distance covered (95% CI 1–24, $\eta_{{\text{p}}}^{2}$ = 0.19, *p* = 0.037, *d* = 0.46, Fig. 2a). This result was independent of sex (women − 5 ± 21 m (− 0.6%); men − 17 ± 30 m (− 1.7%), $\eta_{{\text{p}}}^{2}$ = 0.04, *p* = 0.36), although the women also covered less distance than men (men: 961 ± 12 m vs. women: 856 ± 4 m, $\eta_{{\text{p}}}^{2}$ = 0.23, *p* = 0.021). There were some differences in pacing strategy between sexes, with women taking a more even approach and men displaying negative pacing (Fig. 2b, c), but there were no distinct periods in the trial where pacing was affected by the use of an HME.

Exploration of factors associated with the magnitude of performance decline with HME revealed a significant association between body mass (but not absolute or relative *V*O_2peak_, *V*_Epeak_, sex, or age) and the effect of the HME. Individuals with higher body mass showed a greater negative effect of the HME, when controlling for distance covered in the test without HME (*r*^2^ = 0.22, *p* = 0.027).

There were no differences in post-TT RPE with and without HME (HME: 18.5 ± 1.2 vs. no-HME: 18.4 ± 1.2; $\eta_{{\text{p}}}^{2}$ = 0.05, *p* = 0.28). Neither did lactate accumulation (∆La^−^) differ with and without HME across the cohort; however, there were sex differences in ∆La^−^ between trials ($\eta_{{\text{p}}}^{2}$ = 0.25, *p* = 0.019). The female participants accumulated more ∆La^−^ while performing the TT with HME (HME: 9.3 ± 3.1, no-HME: 7.8 ± 1.4 mmol·L^−1^, *n* = 7, *p* = 0.044), whereas there were no differences in ∆La^−^ among the male participants between trials (HME: 7.9 ± 2.7, no-HME: 8.6 ± 2.8 mmol·L^−1^, *n* = 15, *p* = 0.18).

Across the cohort, the difference in distance covered between TTs (∆dist: distance with HME – distance without HME) was positively associated with both the difference in RPE (∆RPE; *r*^2^ = 0.29, *p* = 0.008) and lactate accumulation (∆La^−^; *r*^2^ = 0.28, *p* = 0.011). At the *y* intercept for each model (equal RPE or equal lactate accumulation, respectively), performance change was negative but with wide confidence intervals (intercept (95% CI): *x*, ∆RPE = 0, *y*, ∆dist = − 19 (− 53 to 3) m; *x*, ∆La^−^ = 0 mmol·L^−1^, *y*, ∆dist = − 13 (− 44 to 16) m).

### Effect of the HME on physiological responses to the TT

**Fig. 3:**
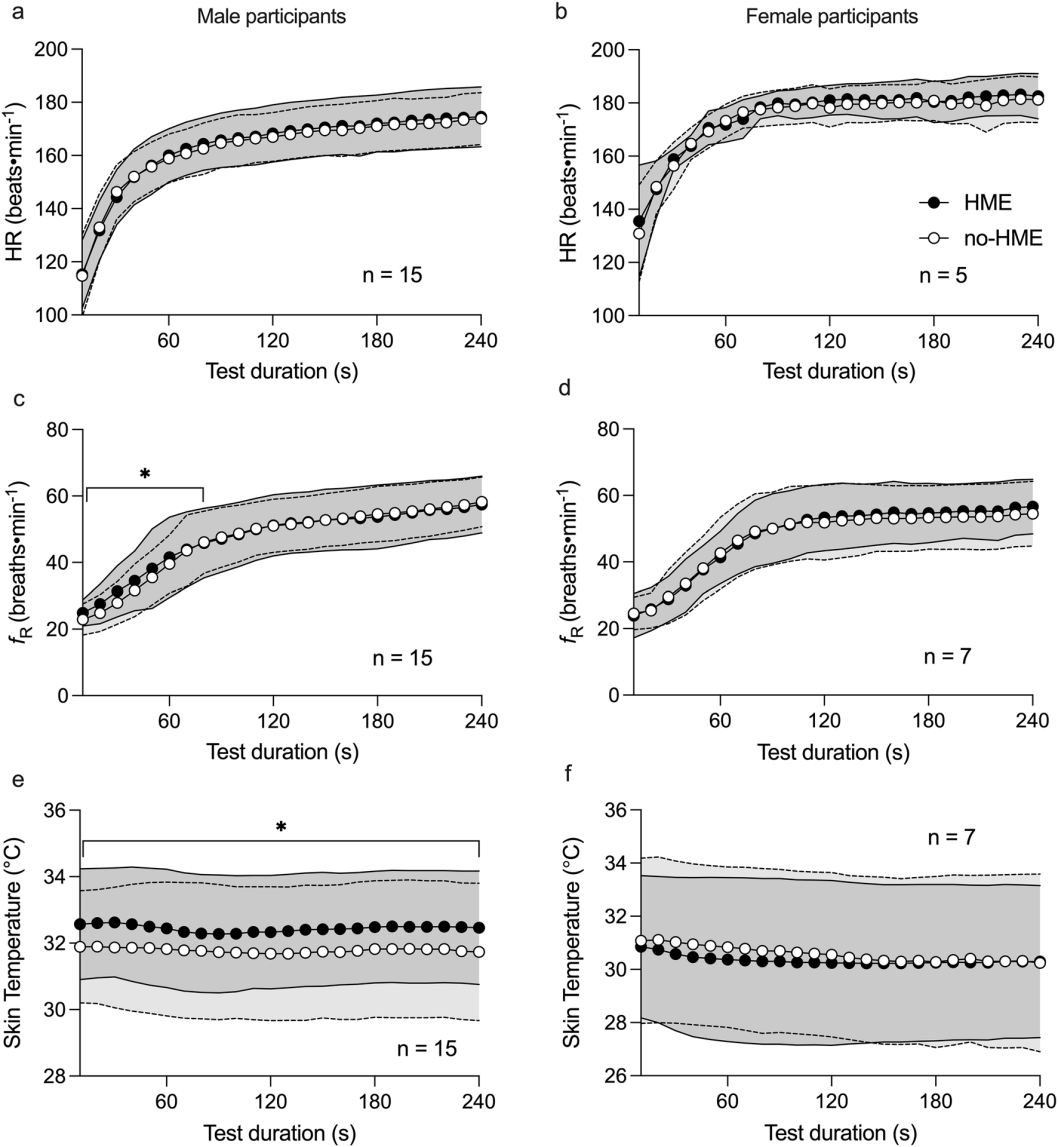
Physiological responses to the 4 min TT for male and female participants. Heart rate (HR) in male (**a**) and female (**b**) participants; respiratory frequency (*f*_R_) in male (**c**) and female (**d**) participants and left lateral thoracic skin temperature (*T*_sk_) in male (**e**) and female (**f**) participants. Data points represent means and shaded bands SD; HME: mid grey and solid lines; no-HME: light grey and dotted lines. *Difference between trials at specified time points, *p* < 0.05

Use of the HME did not influence HR during the TT (HME: + 0.91 (95% CI − 0.26 to 2.08) beats·min^−1^, p = 0.13) (Fig. 3a, b). Neither was there an overall effect of the HME on *f*_R_ (HME: + 0.55 (95% CI − 0.24 to 1.36) breaths·min^−1^, *p* = 0.17). However, *f*_R_ responses during the TT with and without HME differed between sexes (*p* = 0.012). For the women, *f*_R_ did not differ between trials (Fig. 3d), whereas for men, *f*_R_ was higher during the first 80 s of the HME trial compared to without HME (Fig. 3c). *T*_sk_ was higher during the TT with HME in the whole cohort (*p* = 0.001), but a trial × sex interaction (*p* = 0.01) revealed that *T*_sk_ was only higher during the TT with HME in male participants (HME: + 0.66 (95% CI 0.51–0.81), *p* < 0.001; Fig. 3e).

**Fig. 4:**
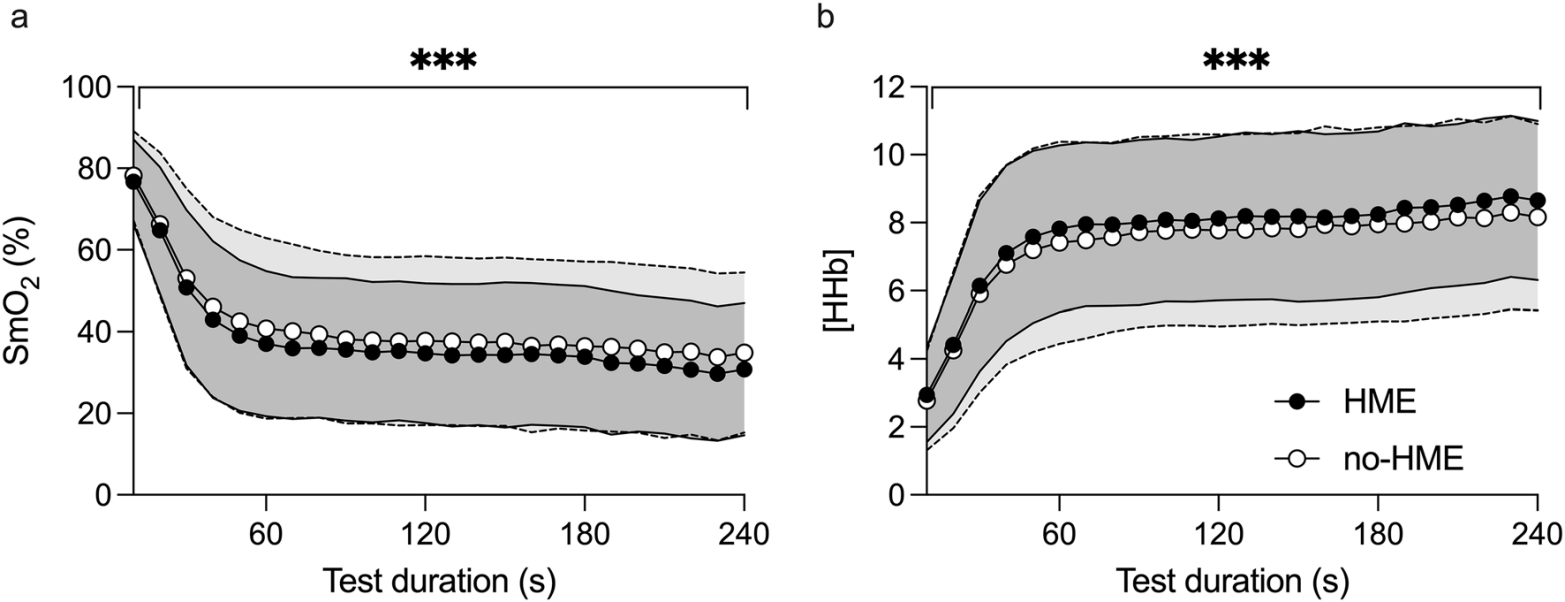
Muscle oxygenation (SmO_2_, **a**) and relative deoxyhaemoglobin concentrations ([HHb], **b**) from *n* = 20 participants during the 4-min running time-trial. Data points represent means and shaded bands SD; HME: mid-grey and solid lines; no-HME: light grey and dotted lines. ***Difference between trials, *p* < 0.001

Muscle oxygen saturation (SmO_2_) was lower during the HME trial versus no-HME (− 3.1%, 95% CI − 2.7 to − 3.4%, *p* < 0.001; Fig. 4a) and lower in men than women (− 20%, 95% CI − 34 to − 6%, *p* = 0.009). [HHb] was also higher in the HME trial vs. no-HME (0.35 arb. unit, 95% CI 0.31–0.39 arb. unit, *p* < 0.001; Fig. 4b) and higher in men than women (3.0 arb. unit, 95% CI 1.1–4.9 arb. unit, *p* = 0.006). Peak deoxygenation (maximum to minimum during the TT) was − 49 ± 12% in the HME trial versus − 47 ± 15% in the no-HME trial (*p* = 0.10).

### Effect of the HME on physiological demands of submaximal exercise

**Fig. 5:**
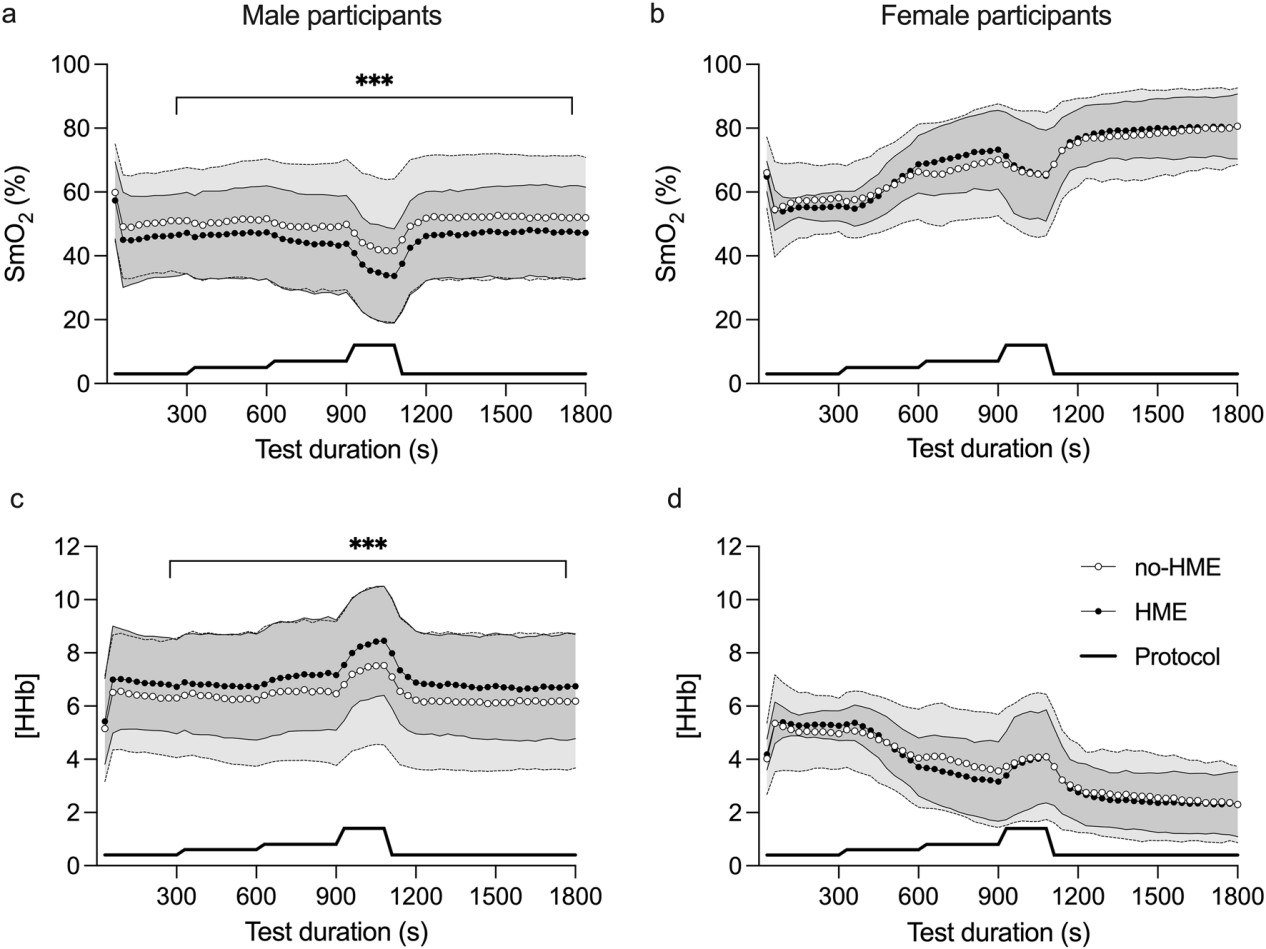
Muscle oxygenation (SmO_2_, **a** and **b**) and relative deoxyhaemoglobin concentration ([HHb], **c** and **d**) in *n* = 12 male and *n* = 7 female participants. Data points represent means and shaded bands represent SD; HME: mid-grey and solid lines; no-HME: light grey and dotted lines. Data used for analysis were 60 s averages taken 90–30 s before the end of each stage. Solid lines at the bottom of each diagram illustrate the varying submaximal exercise intensities. ***Significant difference between trials, *p* < 0.001

During SUB, HR was 2.7 beats·min^−1^ higher during the HME trial (95% CI 1.8–3.6 beats·min^−1^; *p* < 0.001, Table 2). There was no significant difference in *f*_R_ between SUB bouts during the two trials. *T*_sk_ was 0.34 °C higher with HME during SUB (95% CI 0.02–0.66 °C, *p* = 0.04, Table 2), with no significant sex differences. There was a sex × trial interaction (*p* = 0.05) as well as a main effect of HME on RPE (*p* = 0.02, Table 2); post hoc analysis revealed that RPE was higher during the HME trial, but only in the women (women: 0.65, 95% CI 0.17–1.13, *p* = 0.008; men: 0.05, 95% CI − 0.30 to 0.41, *p* = 0.77). There were also trends towards overall effects of the HME on SmO_2_ and [HHb] (*p* = 0.051 and *p* = 0.091 respectively). However, the effect of the HME on SmO_2_ and [HHb] during SUB varied with sex (*p* = 0.007 and *p* = 0.009; Fig. 5). Among the male participants only, SmO_2_ was lower during the HME trial (− 3.8%, 95% CI − 1.9 to − 5.6%, *p* < 0.001; Fig. 5b), while [HHb] was higher in HME (0.42 arb. unit, 95% CI 0.20–0.65 arb. unit, *p* < 0.001; Fig. 5d).

**Table 2:** Heart rate (HR), respiratory frequency (*f*_R_), left lateral thoracic skin temperature (*T*_sk_), and Borg rating of perceived exertion (RPE) during each of the five stages in the submaximal exercise bout

		65% *V*O_2peak_ 3:30–4:30	70% *V*O_2 peak_ 8:30–9:30	75% *V*O_2 peak_ 13:30–14:30	90% *V*O_2 peak_ 16:30–17:30	65% *V*O_2peak_ 28:30–29:30	Main effect of HME	HME × stage interaction
HR, beats·min^−1^								
HME		134 ± 12	144 ± 12	150 ± 12	164 ± 12	140 ± 11	***p < 0.001***	*p* = 0.87
No-HME		132 ± 12	141 ± 11	147 ± 11	161 ± 11	137 ± 10		
*f*_R_, breaths·min^−1^								
HME		32 ± 6	36 ± 6	37 ± 7	42 ± 7	37 ± 7	*p* = 0.89	*p* = 0.69
No-HME		31 ± 7	35 ± 7	38 ± 8	42 ± 8	37 ± 8		
*T*_sk_, °C								
HME		32.2 ± 1.2	31.7 ± 1.7	32.1 ± 1.9	32.4 ± 1.9	32.8 ± 2.3	***p = 0.04***	*p* = 0.99
No-HME		31.9 ± 1.0	31.5 ± 1.5	31.7 ± 2.0	32.0 ± 2.1	32.4 ± 2.5		
RPE								
HME		9.5 ± 1.6	11.7 ± 1.5	13.3 ± 1.8	15.4 ± 1.8	10.3 ± 1.7	***p = 0.02***	*p* = 0.50
No-HME		9.3 ± 1.7	11.3 ± 1.6	12.9 ± 1.8	14.9 ± 1.5	10.6 ± 1.7		

Data are male and female participants’ combined, mean ± SD

Bold italic text indicates statistically significant findings (*p* < 0.05)

### Participants’ HME usage and perceptions of the effect of HME on performance

Nine participants (39%) reported that they at least occasionally used an HME when the environmental temperature was below 0 °C, increasing to 14 (61%) participants who at least occasionally used an HME when temperatures were below − 20 °C. There was no difference in participants’ judgement of their performance between trials (Q1 median (range): no-HME = 4 (2–6), HME = 4 (2–6), *p* = 0.54). Neither did the perception of the effect of the HME on performance differ between trials (Q2_C_ = 4 (1–7), Q2_H_ = 4 (4–6), *p* = 0.36). After the no-HME trial, five (22%) participants described airway symptoms/discomfort, whereas two participants (9%) noted that they did not feel they were much affected by the cold environment. After the HME trial, six participants (26%) noted a build-up of humidity, or ice, in the mask, with three (13%) mentioning a build-up of mucus. Six participants (26%) noted that the mask was uncomfortable, unpleasant, or a distraction, three (13%) mentioned a poor fit, and five (22%) said that they felt the HME restricted their breathing. Four participants (17%) mentioned that the mask was comfortable, felt good, or mitigated symptoms, and no participants mentioned airway symptoms or discomfort associated with wearing the mask. One participant described that water dripped onto the treadmill from the mask and froze to form ice, which was a distraction during the TT.

## Discussion

This study was the first to investigate the effects of an HME on exercise performance in healthy individuals. Use of an HME decreased distance covered during a self-paced running TT by approximately 1.4% in a sub-zero environment. Whilst this degree of performance impairment is within the coefficient of variation for a 4-min, self-paced running TT (McGawley 2017), it would equate to a 3.4-s disadvantage over a ~ 4-min competition. Such a margin may make a meaningful difference to performance at an elite level in cross-country skiing; 14 s over a 4-min course have been shown to span 46 places in an international sprint-ski competition (Sandbakk et al. 2011), so 3.4 s could equate to ~ 10 places. In contrast, the previous studies have shown no effect of other, mouthpiece-style HME models on near-maximal exercise capacity in healthy adults, albeit not in a performance setting (Eiken et al. 1989; Frischhut et al. 2020). Improvements to sprint performance have also been demonstrated when a mask-like HME is used between exercise bouts (Seifert et al. 2017).

While there were no significant sex differences in the effect of the HME on performance, the mean distance covered was 0.6 and 1.7% lower with HME in women and men, respectively. Higher body mass was also associated with a greater impairment to performance, and the range of body mass was much greater among the men. The mechanism underpinning this outcome is unclear, as we found no relationships between *V*O_2peak_ or *V*_Epeak_ and performance change with HME. Generally, larger people retain more body heat than smaller people as a result of greater muscle mass (Toner et al. 1986), and because body surface area does not increase proportionately with increased mass. However, the increased *T*_sk_ with HME during the TT in men could suggest that the HME impaired respiratory heat loss during the TT and more readily led to mild hyperthermia in heavier participants, just as breathing cool air has been shown to offset hyperthermia during exercise in hot conditions (Geladas and Banister 1988). The women accumulated more blood lactate during the TT with HME than without, while lactate accumulation among male participants was similar with and without HME. Both results thus indicate a reduced aerobic distance contribution (i.e., the distance attributable to aerobic energy supply) during the TT with HME, with the women able to compensate by increasing the anaerobic distance contribution, as indicated by the higher post-exercise blood lactate accumulation in women. A reduced distance contribution from aerobic energy sources could then be attributed to either a reduced *V*O_2_, an increased gross oxygen/energy cost of running, or a combination of the two factors.

NIRS-derived SmO_2_ and [HHb] provided insight into tissue oxygenation, in the absence of a metabolic cart system, which would have been impossible to incorporate without interfering with both the HME and no-HME conditions. To our knowledge, our study was the first to investigate the effects of HME usage during exercise in the cold on these physiological parameters. SmO_2_ decreases in proportion to increases in exercise intensity and may react faster to exercise intensity changes than heart rate (Born et al. 2017). During the TT, SmO_2_ was reduced and [HHb] increased with HME; the same effects were observed during SUB, but only in the men. These observations indicate either increased tissue O_2_ extraction or reduced delivery during the TT. While core temperature was not measured, cooler body temperature (as indicated by lower *T*_sk_ without HME) should cause a leftward shift of the O_2_ dissociation curve (Barcroft and King 1909), leading to impaired O_2_ unloading at the tissues without HME (or improved unloading with HME). Thus, warmer body temperature (indicated by higher *T*_sk_) with HME correlates with lower tissue oxygenation. However, if increased O_2_ extraction did occur with HME, it did not translate to better performance, suggestion that reduced O_2_ delivery may also have been a determining factor for performance. The relative influence of the approximate 0.1 L HME dead space on inspired gas fractions should decrease as tidal volume increases (Ainegren et al. 2020). However, the higher *f*_R_ with HME in men at the start of the TT may have exacerbated the influence of the HME dead space on inspired gas fractions and thus impaired gas exchange at the lungs.

NIRS signal quality during SUB appeared poorer for the women, and there was also a trend towards increased SmO_2_/decreased [HHb] in female participants throughout the 30-min submaximal warm-up. An explanation for the poorer quality readings at the beginning of the women’s warm-up could be a combination of body composition and metabolic heat production due to the known sex differences in human thermosensitivity to cold air (Graham 1988). It is possible that reduced peripheral blood flow, or reduced metabolic rate, in the early stages of SUB could have negatively affected the NIRS readings in women (Tew et al. 2010); although only one location for *T*_sk_ on the trunk was included, and the NIRS sensors were placed on *m. vastus lateralis*. Although the reliability of the MOXY monitor has been questioned at higher exercise intensities (Crum et al. 2017), the signal in the present study appeared to be of better overall quality during the TT than SUB, after participants were fully warmed up.

Physiological responses during SUB and the TT also provided insight into the influence of the HME. HR was higher with the HME during the fixed-workload submaximal exercise, but not different in the TT. Considering that distance covered was reduced with HME, this indicates increased cardiovascular strain at a given running speed with HME. This finding contrasts with that of Frischhut et al. (2020) where HR trended lower during high-intensity exercise with HME. Other than an elevated *f*_R_ during the first 80 s of the TT among male subjects, *f*_R_ was not affected by HME. To our knowledge, no previous literature has investigated the effects of an HME on *f*_R_ nor how it may relate to exercise performance. As *V*_E_ will be affected by rate, depth, and resistance to breathing, so *f*_R_ reflects only one part of the overall influence of the HME on ventilation. Based on recent evidence, it is unlikely that the increased *f*_R_ would have increased resistance to breathing in the HME filter, because the resistance in the filter used in the present study should equate to a very low energy cost, even at higher *V*_E_ (Ainegren et al. 2020). In the current study, *f*_R_ was measured, since it is non-invasive and should not have interfered with the HME condition, and, additionally, *f*_R_ has been reported to correlate with perceived exertion (Nicolò et al. 2017). As perceived exertion was only documented after the TT, to avoid distracting the participant from the performance test, *f*_R_ could be considered a proxy measure of perceived exertion throughout the TT.

RPE was higher during SUB with HME in the women, but unaffected by HME usage in the TT, suggesting a similar perceived exertion was achieved in both maximal TTs. However, considering that performance during the TT was decreased, it could be interpreted that the HME increased perceived exertion at a given near-maximal running speed. These findings are contrary to previous research, where HME usage decreased RPE in healthy winter athletes during maximal exercise in − 20 °C (Frischhut et al. 2020). However, given that pacing strategy was not altered during the TT with HME, and participants rated their performance similarly on both TTs, the HME probably did not introduce specific perceptual barriers to performance. Moreover, small differences in distance covered seemed difficult to perceive. Responses to our questionnaire suggest that several participants were accustomed to using HMEs during training in sub-zero conditions, potentially to improve respiratory comfort, or as a prophylactic against airway injury or bronchoconstriction.

Many of our findings are contrary to those of the only known previous study of HME effects on physiological and perceptual variables during high-intensity exercise (Frischhut et al. 2020). However, the two study protocols differ in the type of HME used (mouthpiece vs. mask), exposure duration (24 vs. 45 min), temperature (− 20 vs. − 15 °C), and exercise protocol (fixed pace with external modification if needed, vs. self-paced), intensity (fixed, high-intensity vs. graded submaximal then maximal TT), and duration (8 vs. 30 + 4 min), and thus, differences in results between the two studies should be interpreted with caution. A potential advantage of the HME utilised in the present study is that the semi-flexible foam mask covered the mouth and nose, creating a dead space for warm, humid air to accumulate. However, the material also allowed for substantial ice build-up in the filter from the accumulated moisture, which may restrict airflow, and 39% of the participants noted accumulation of mucus and humidity in the mask. In contrast, Frischhut et al. utilised a mouth-held HME that did not provide any coverage of the face. In the latter study, 85% of the participants reported excessive mucus build-up in the HME, and 23% reported gum pain. Conversely, a higher proportion of participants reported an improvement in comfort with the HME compared to the present study (77 vs. 17%). Therefore, there are comparative advantages and disadvantages to different HME designs, not only in function, but also in user comfort and/or preference, and potential effects on performance. It is possible that the increased dead space with the mask compared to a mouthpiece-like device may have led to increased rebreathing of expired gases, leading to impaired gas exchange and a poorer performance outcome.

While there is some variation in HME design (Ainegren et al. 2020), the mask-like HME in the present study was selected based on having low resistance to breathing (Ainegren et al. 2020), being frequently used by Swedish cross-country skiers (Stenfors et al. 2020), and providing partial coverage of facial skin, and thus, we expected it to have a good likelihood of being beneficial for performance. Several participants in the present study did perform better with the HME than without, but the comments from these individuals did not always correspond to the performance outcome (e.g., reporting relief from cold-induced symptoms with HME, but still performing worse). While highly anecdotal, and keeping in mind that there will always be day-to-day variation in performance, these observations may also serve to highlight that subjective experiences may not necessarily align with actual performance outcomes, and are also in line with the previous studies that demonstrate substantial inter-individual sensitivity to cold air (Beuther and Martin 2006; Kennedy et al. 2019). Thus, the likelihood of heterogeneous responses and need for objective testing of HME effects are worth considering if providing guidelines regarding HME use to individual athletes.

Realistic temperatures for cross-country skiing training and competitions range from − 20 °C to above 0 °C (Sandsund et al. 2012). The environmental chamber delivered a stable temperature with a slightly lower relative humidity than would be expected in an outdoor environment at − 15 °C in Sweden (Wern 2013). A weakness in the experimental set-up was the use of a running protocol, as opposed to roller-skiing, which was a necessary compromise to deliver a controlled exercise protocol in a sub-zero environment. However, it is not uncommon for studies focused on cross-country skiing to employ running protocols in a laboratory environment (Sandsund et al. 2012; Frischhut et al. 2020) and the uphill gradient was introduced to minimise the risk of a neuromuscular limitation to performance (Paavolainen et al. 2000). One participant commented that water dripped from the HME and froze on the treadmill, which may have introduced an artificial confounder that could have distracted participants during the performance test. A further weakness of the present study was the lack of a placebo or sham condition, which has been incorporated in the previous studies of HME use during exercise (Beuther and Martin 2006; Jackson et al. 2020), but these studies also suggest that a true placebo is difficult to achieve without introducing any extra dead-space that can serve as pre-station for heat exchange, and is arguably not an ecologically valid intervention.

The present investigation included participants from a wide range of performance levels, from physically active adults to elite athletes. The participants were also not all cross-country skiers, though all were accustomed to endurance training in the winter. Without stratifying data by performance level, age, training status, or aerobic capacity, it is difficult to make specific recommendations for athletes. However, the broad range of physically active subjects in this study did allow us to investigate the relationship between fitness and the effect of the HME on performance. Given we found no relationship between *V*O_2peak_ and the effect of the HME, our findings appear to be valid across a range of performance levels. This may provide initial information to both elite and recreational-competitive athletes in their decisions as to whether to use an HME during competition.

The findings of this research invite further investigation of the effects of HMEs on lung function and physiology in non-asthmatic individuals. As highlighted by Seifert et al. (2017), it may also be relevant to investigate the effects of using an HME on performance when applied during the warm-up only. Investigation of HME effects on performance, physiology, and comfort over longer training and competition durations is also warranted, given that cross-country skiing competitions may last over 2 h for a ≥ 50-km event in sub-zero conditions. Most importantly, research is lacking regarding potential prophylactic effects of HMEs on the development of exercise-induced asthma from training in cold climates.

## Conclusions

Among healthy individuals, use of a mask-like HME decreased distance covered in a self-paced running TT by approximately 1.4% in a sub-zero environment. Despite a reduction in performance, pacing strategy, HR, perceived exertion and lactate accumulation were not affected by the HME during the TT, but reduced SmO_2_ and increased [HHb] indicated that tissue oxygen delivery was reduced and/or extraction increased while using an HME. *T*_sk_ was also higher during the TT with HME in men, indicating that the HME may have inhibited respiratory heat loss. At submaximal workloads, HR and *T*_sk_ were higher with HME, and tissue oxygenation was reduced among men. Taken together, our findings suggest that an HME impairs performance and increases the physiological demands of maximal and submaximal exercise, particularly in men and individuals with higher body mass. The reasons for the scarcity of elite athletes using HMEs in competition are unclear, but the present study highlights that an HME can negatively affect healthy individuals’ performance in sub-zero conditions, to an extent sufficient to alter competition outcomes.

## Data Availability

The datasets presented within are not publicly available, because consent for this was not obtained from the participants nor approved by the ethical review board. However, the data are available from the corresponding author upon reasonable request.

## Abbreviations

ANOVA: Analysis of variance
Arb. unit: Arbitrary units
CI: Confidence interval
CV: Coefficient of variation
dist: Distance covered during the TT
EIA: Exercise-induced asthma
EIB: Exercise-induced bronchoconstriction
FEV_1_: Forced expiratory volume in 1 s
*f*_R_: Breathing frequency
HME: Heat-and-moisture-exchanging breathing device
HR: Heart rate
La^−^: Blood lactate concentration
NIRS: Near infra-red spectroscopy
RERpeak: Peak respiratory exchange ratio
RPE: Borg 6–20 rating of perceived exertion
SmO_2_: Muscle oxygenation
SUB: Submaximal exercise bout
*T*_sk_: Skin temperature
TT: Four-minute, self-paced, treadmill running time-trial
*V*_E_: Minute ventilation
*V*_Epeak_: Peak minute ventilation
$$\dot{V}{\text O}_{2}$$: Oxygen consumption
$\dot{V}{\text O}_{2peak}$: Peak oxygen consumption
[HHb]: Relative deoxyhaemoglobin concentration
[THb]: Relative total haemoglobin concentration

## Code availability

The Python code that was used for physiological data preprocessing is available on request.
